# P04.45. A systematic review of randomized clinical trials on herbal medicines for treatment of fatty liver diseases

**DOI:** 10.1186/1472-6882-12-S1-P315

**Published:** 2012-06-12

**Authors:** Z Liu, Y Fei, G Li, S Grant, B Roufogalis, J Liu

**Affiliations:** 1 Beijing University of Chinese Medicine, Center for Evidence-based Medicine, Beijing, China; 2 Faculty of Pharmacy, The University of Sydney, Sydney, Australia; 3 University of Western Sydney, Sydney, Australia

## Purpose

Assess the beneficial and harmful effects of herbal medicines on Fatty Liver Disease (FLD).

## Methods

We searched for randomised clinical trials comparing herbal medicine with placebo, no treatment, lifestyle therapy or western interventions in participants with FLD in The Cochrane Hepato-Biliary Group Controlled Trials Register, The Cochrane Library, MEDLINE, EMBASE, Science Citation Index Expanded till May 2011, Chinese BioMedical Database, Traditional Chinese Medical Literature Analysis and Retrieval System, China National Knowledge Infrastructure, Chinese VIP Information, Chinese Academic Conference Papers Database, Chinese Dissertation Database, and Allied and Complementary Medicine Database till June 2011. We used risk of bias to assess trial methodological quality. The effects estimates were presented as relative risks (RR) with 95% confidence intervals (CI) or as mean differences (MD) with 95% CI depending on variables of the outcome measures.

## Results

We included 25 randomised trials which involving 2257 FLD participants. The mean sample size was 90.3 participants (ranging from 40 to 146 participants per trial). Risk of bias of the included trials was high or unclear. Twenty-three different herbal medicines were tested in the RCTs. None of the trials reported death, hepatic-related morbidity, quality of life or cost. Surrogate outcomes such as serum AST, ALT, GGT, ALP, B ultrasound findings, CT scan findings and adverse effects were reported. Qinyin tea and Danning tablet did not show beneficent effects on outcomes of ALT, AST, B ultrasound and ALP. Qinyin capsule did not show beneficial significant effect on ALT (MD 17.80, 95% CI 6.35 to 29.25). Other herbal medicines showed beneficial effect on at least one of the reported biochemical outcomes. No serious adverse events were reported.

## Conclusion

Some herbal medicines may have potential effects on FLD and they appear safe to use. The findings are not confirmatory due to high or unclear risk of bias of the included trials and limited number of trials on individual herbal medicines.

